# Bereaved Families: A Qualitative Study of Therapeutic Intervention

**DOI:** 10.3389/fpsyg.2022.841904

**Published:** 2022-02-28

**Authors:** Valeria Moriconi, María Cantero-García

**Affiliations:** ^1^Hospital Universitario Infantil Niño Jesús, Fundación Aladina, Madrid, Spain; ^2^Department of Psychology, Faculty of Biomedical and Health Sciences, Universidad Europea de Madrid, Madrid, Spain; ^3^Department of Psychology, Faculty of Health Sciences, Universidad Internacional de Valencia, Valencia, Spain

**Keywords:** grief, bereaved parents, psycho-oncology, group therapy, pediatric oncology, meaning-making, legacy

## Abstract

**Background:**

A child’s death is the most stressful event and the most complex grief that families face. The process of psychological adaptation to the illness and death of a child is difficult due to a variety of emotional reactions. Parental grief had received the attention of researchers only in recent years when it became clear that this reality differs substantially from the general grief process.

**Objective:**

This work aims to highlight the needs of bereaved parents; increase the specificity and effectiveness of the therapeutic approach to prevent complications in the process of loss-making; and find the recurrent thematic nuclei in the development of bereavement present in a therapeutic group of parents who have lost their child to an onco-hematological disease.

**Method:**

Between 2011 and 2016, five therapeutic groups for the grief elaboration were made. The sample included a total of 50 parents of children who died from cancer between the ages of 0 and 21 years.

Content analysis was carried out as a qualitative analysis method. The SAS^®^ Text Miner software (SAS Institute Inc, 2004) was used to read, interpret, classify and integrate the data from numerous sources.

**Results:**

The development and consecutive interpretation of the 5 clusters have been carried out to analyze the related topics using the node “Topic Analysis” and requesting the subdivision into five topics. Four topics have been well defined. Clear topics are reducible to categories of emotional *relief, tools, legacy, and unfinished business*. The topic analysis provides interesting indications about the different interpretive journeys of the bereavement situation and offers ideas regarding the different types of social responses.

**Conclusions:**

After reviewing the existing bibliography, we have confirmed the lack of specific literature on the problem of grief in parents whose children have died from cancer. Much research has shown that parents who lose a child to cancer want support, and there are still few studies on the most effective interventions for this group.

## Introduction

The death of a child is considered taboo, underlining this type of loss’s paradoxical and contradictory nature (Barbero and Alameda, 2009). The process of psychological adaptation to the disease, the death process, and the grieving process over the death of a child is a very complex stage for professionals and the family due to the variety of emotional reactions that happen (Die-Trill, 2003).

The specificity of bereavement in parents has been highlighted by Rando (1998) as an explanatory model different from other types of grief. Rando observes that these factors are as follow: premature loss; quality and nature of the relationship with the deceased, role of the deceased, death characteristics, and quality of the supporting social network. They are grouped to form a characteristic constellation, which explains the depth of the wound suffered by the parents. The death moment is necessary to highlight the importance of both the common aspects and the differential variables in mourning a child’s death (Field et al., 2013). Studies have shown that bereavement in parents usually persists and often intensifies, even after the first year after death and is associated with higher Prolonged Grief Disorder severity compared to loss of a parent or a sibling (Lichtenthal and Breitbart, 2015; Zhou et al., 2020).

Tizón and Sforza (2008) says that parents feel that an essential part of them dies when a child dies. “The most important bond of love is affected: love for life itself.” Parents lose their child and what their child represents, a perspective toward the future, offspring, dreams (Schiff, 1997; Talbot, 2002; Zheng et al., 2017). The death of a child subverts the natural order of events, being experienced as illogical and against nature (Davies, 2004).

Parental grief has received the attention of researchers only in recent years when it became clear that this reality differs substantially from the general grief process. Different studies (Tan et al., 2012; Zhou et al., 2018; Morris et al., 2019) demonstrate the need to develop specific explanatory models for this phenomenon. Families face the difficult job of building a new world of beliefs and values where the reality of loss can take hold, and this process takes months and years to be complete, even knowing that it may never be resolved. This line understands the importance of providing effective interventions to reduce the long-term complications that may manifest in bereavement (Richardson et al., 2011; Wiener et al., 2019; Kochen et al., 2020).

We are working with bereavement parents in a therapeutic group setting. The group becomes an instrument of change by providing members opportunities to work and talk about the problems in their lives. Studies are still very scarce and often inconclusive, demonstrating the need to explore this methodology and this well-defined theme further.

This work aims to highlight the bereaved parent needs; increase the specificity and effectiveness of the therapeutic approach to prevent complications in the process of loss-making. Find the recurrent thematic nuclei in the development of bereavement present in a therapeutic group of parents who have lost their child to an onco-hematological disease.

## Materials and Methods

### Setting and Recruitment

Between 2011 and 2016, five therapeutic groups for grief elaboration were made. The sample included a total of 50 parents of children and adolescents who died from cancer. The population comes from the “Niño Jesús” University Children’s Hospital in Madrid and the Gregorio Marañón General University Hospital. Therapeutic intervention is developed by a therapist who is an expert in psycho-oncology and bereavement at the Aladina Foundation Psychological Care Center. This independent organization dedicates its efforts to cancer children and adolescents. Participants are contacted by a therapist approximately 2 months after the death of their child. Before the start of group therapy, the participants carry out several individual or couple interviews with the therapist. The objective is to sign the informed consent to guarantee the privacy and the use for educational purposes of the data collected during the sessions.

The inclusion criteria for group therapy were (1) being the parents of a deceased child in pediatric age (up to 21 years) treated for an onco-hematology disease; (2) being in grief in a time between 2 months and a year; (3) compliance with group norms of acceptable behavior; (4) motivation to do bereavement work; and (5) not participating at the same time in other group psychological therapies.

The exclusion criteria were (1) inability to tolerate the group setting; (2) tendency to assume a deviant role; (3) disagreement with group standards of acceptable conduct; (4) serious incompatibility with one or more members of the group; (5) lack of motivation to work.

### Analysis Data

The *Content Analysis* of transcripts of therapeutic group sessions was used for a qualitative study. The objective of content analysis is “to provide knowledge and understanding of the phenomenon under study” (Downe-Wamboldt, 1992). The SAS^®^ Text Miner software (SAS Institute Inc, 2004) was used to read, interpret, classify and integrate the data from numerous sources. The cluster analysis process consists of three steps.

### Cluster Analysis

#### Phase I: Transcript of the Sessions

The basis from which the data were extracted for the discourse analysis was the transcripts of psychotherapeutic sessions. The therapist wrote the phrases of each patient verbatim. At the end of each session, the notes were reviewed, and the transcription expanded. Transcripts of the interventions of mothers and fathers were analyzed in depth. Recording what they said, and how they expressed it, avoiding modifying their language. Consequently, some fragments appear as grammatical forms and others as colloquial forms.

#### Phase II: Processing Data

The transcripts, 111 files in total, were prepared in table format to be analyzed by the software. The analysis “Text Parsing” allows the analysis of a set of documents to quantify the information about the terms in it, generating a frequency matrix of the words in the papers. In Text Filtering, the Log function has been used to calculate the weight of the frequency to associate for each term. Attributing weights to words is helpful to distinguish important terms from others. Successively two weighting flows have been created, the “inverted frequency”; in the second one the “entropy.” The “inverted frequency” method gives more weight to terms that infrequently occur in the sample of documents by placing the number of records that contain the word in the numerator of the formula. While the “entropy” method gives greater weight to terms that infrequently occur in the sample of documents using a derivative of the entropy measure found in information theory. Once the results were obtained, it was decided to consider the second flow (entropy) for the construction of the topics since it reflected better in terms of the categories obtained.

The Text Filtering and Document Analysis nodes must identify the set of valid terms of which we have filtered 214 definitive analysis units. With the 214 units, we have carried out a hierarchical Cluster Analysis to understand what the ideal partition of the categories could be. We have suggested a division of the data into five clusters.

#### Phase III: Topic Analysis

The realization and consecutive interpretation of the 5 clusters have been carried out to analyze the related topics using the node “Topic Analysis” requesting the subdivision into five topics. Since the results of this analysis, four topics have been well defined while another is too heterogeneous. Clear topics are reducible to categories of emotional *relief, tools, legacy, and unfinished business*.

The first flow of analysis using the inverted frequency had led to the recognition of a single well-defined topic that could be retransmitted to the category “sense.” Considering this data, we have used the terms with the most significant weight of this topic to manually define five topic knot Text Analysis of the second flow. To further verify the results, the words with the most important weight of the topic “meaning” have been measured in the other four topics, and it has been seen that they have a low weight. Therefore, the creation of a different category is justified. With the insertion of the topic defined by inverted frequency, the node has been re-executed, and five well-defined and representative topics have been delineated for the research.

## Results

The first topic called “meaning” has been defined by 69 words present in 21 documents. The second topic called “emotional relief” has been described by 27 words present in 16 documents. The third topic called “legacy” has been defined by 17 words present in 14 documents. The fourth one called “tools” has been defined by 31 words present in 11 documents. The fifth one called “unfinished business” has been defined by 31 words present in 19 documents (Table 1).

**TABLE 1:** Topic analysis.

Topic	Topic description	Number of words	Number of files
Birthday, death, fear, sadness, feeling, living	Emotional relief	27	16
Qualities, understanding, desire, support, person	Tools	31	11
Guilt, accept, why, time, understand	Pending issues	31	19
Legacy, love, life, contact, experience	Legacy	17	14
Contribute, happy, spirituality, tribute, channel	Meaning	69	21

The associations, which each word has with the others, are analyzed through concept linking diagrams that show the frequency with which one word appears next to another. We decided to investigate the words that are not repeated in previous connections and underline the words with closer relations with the central concept to give a broader perspective of the topics.

### Sense

We will describe the words to define this topic: contribute, happy, spirituality, tribute, and channel. Internal schemas protect against non-sense, and once loss knocks them down, the emptiness remains. If the emptiness appears as deep pain on an emotional level, on a cognitive level, the emptiness seems non-sense. The word meaning refers to the grieving people’s attempt to seek meaning in their pain experience. In this process, mourners discover that the loss can lead to personal growth, such as a new perspective or learning something improved about themselves.

Parents feel the need to contribute to people, to turn their experiences into teachings or examples for others. They have learned how to deal with difficult times; they feel proud of their children and themselves for having enriched themselves with this experience and for having collapsed.

***“Thanks to my daughter and everything that has happened, I know how to say no, I know how to live without anyone conditioning me, and I do not prepare anyone. I have changed my values; I give importance to what has it, I express my feelings more.”***

Happiness is felt again, with the premise that you can no longer be happy as before, and you must build another way of being happy.

***“I feel fortunate to have been able to meet you and teach me the purest and most eternal love that will ever exist, mother-child love.”***

When death ceases to be scary and is integrated into everyday life, a connection is felt with another dimension, a spiritual dimension that should not be seen only as a call to religion but as a greater goal.

***“I want to help others; I want to show more intensity for my own, I want to say I love you more times with more value, I recognize the importance of health, taking care of you, I have learned to say no, I am more spiritual.”***

Mourners try to give a new meaning to their lives by living it as a tribute to their children. They want to show that their children have transmitted their courage, the same with which they have faced illness and death. Parents cannot leave this learning in a vacuum since it is already part of them. Thus, they rescue it and teach it.

***“This whole situation made me stronger, braver, more fighter, and taught me not to let minor problems destroy me. My daughter V. came into the world to make me a better person and give me a great life lesson.”***

### Emotional Relief

The words that we will describe to define this topic are birthday, death, fear, sadness, feeling, living.

The word “birthday” synthesizes, on the one hand, memories, death, everyday life, friendship, the other, peace, spirituality, and positivity.

Dates such as birthdays, the anniversary of death, and many others that come out of everyday life sharpen emotions in an ambivalence between a feeling of peace, when contacting the memory of the son, or of agony if it is related to the absence, with death.

***“The birthday went well because it is a good day to remember and celebrate, his birth is celebrated, the other anniversary reminds you of his death.”***

Fear is a very common feeling, and at this time of therapy, it is related to contact emotions, opening to this world in which pain and sadness predominate, in which an existential emptiness encompasses everything. The fear of developing depression is present, as well as the fear of never leaving this melancholic state and not knowing how to continue living. Fear serves as a sentinel and an emotion block, which is why it is so important to work with it. Furthermore, facing fear serves to be able to start feeling. Another recurring fear is the fear of forgetting; parents feel that if they stop suffering, feeling sorry, crying, being angry, they lose the essence of their children, lose contact, and forget them.

***“I am very afraid to forget, fear of not being able to approach this pain in another way, fear of never being able to have a close person and give support to those who suffer because it makes me feel too sorry; fear of rejecting all this so as not to feel again.”***

The first emotion that dominates in grief is sadness, huge, deep, portentous, which envelops absolutely everything and never seems to placate. One way of how sadness is expressed is the veto to enjoyment, to happiness. An unconscious treatment is made in which, in memory of the son, any form of well-being is prohibited, and if it occurred, it would be like a betrayal. And it is then that the guilt appears.

***“I do not want to condemn myself and my family to a state of permanent sadness.”***

“Feel” is the core of emotional release. After beginning to express experiences and memories, leaving aside the inner monolog, and opting for a shared verbalization, fears appear. These are the first defense mechanisms that help us not to feel since we cannot feel when we are not prepared. A dichotomy opens when you outline the possibility of returning to life. The energy of life moves the death, the desire to take advantage of life along with the things that are worth, removed fear.

***“Thanks to the fear of losing everything, now I am going with my family instead of doing more hours at work.”***

### Legacy

It is important to be able to create bonds that connect the deceased person and return the bond, which helps elaborate the loss. The important thing is to realize how we need to put what has been lost in a good place in order to light us up and light up to keep walking, instead of clinging to its memory anchored in the past or trying to bury it. It is important to give a new internal place rather than fear or seek oblivion. The words that define this topic are legacy, love, life, contact, experience.

The legacy they leave is an immense love that teaches them what the true way forward is.

***“I want to live from day to day. I have stopped making sense of having things and making plans, now the values are different.”***

The parallelism between life and anguish has changed, and the look to life with strength prevails. Parents continue toward a new connection to life, from the love that makes them feel proud and strong.

***“Now I see life differently, I take advantage of every moment to follow my needs, there is no future project in the heavy sense of responsibility, I enjoy the day to day.”***

Throughout the therapy, new ways of contacting the children other than through grief are learned. We can see now how the fear of letting go of grief.

***“Since my daughter died, I have felt a lot of peace this has made me understand that my daughter was going to stay with me, I don’t want that thinking about my daughter carrying pain and sadness because she does not deserve it, I will always be happy to have had a daughter and to be able to think of her.”***

### Tools

The intensity of pain and suffering is enormous, so parents need to defend themselves. Within limits, these defensive maneuvers in grief can be useful. There is a need to defend yourself to “adapt” to the reality of loss, in any way that everyone is able.

The words described to define this topic are qualities, understanding, desire, support, person.

The word “Qualities” emphasizes self-knowledge and knowledge of others, assuming this material as a resource.

***“I have discovered these qualities. to be understanding and able to listen and talk about pain, love, patience.”***

Mourners face new thoughts and sensations, some heartbreaking, others embarrassing. In group therapy, there is a climate of respect and trust that favors an expression without judgment of which understanding is the fundamental axis.

***“I just want to share how I feel about who understands him like you.”***

Discovering denied parts, validating internal resources, sharing with the group doubts, questions, blocks and achievements is necessary to regain the desire to face life, to seek other ways of living and to recognize and face problems and emptiness.

Emptiness is something that everyone feels in front of the loss of a son or daughter. It is also something that must be defended while it cannot be sustained.

***“I have also found many people who love me; love for others is where I want to start, now I am looking for the good. I have to move forward. And the group helps me because I share, because I know other ways to carry out grief. I feel good.”***

Parents feel support, so they can move forward in their ways and find themselves as people, not only as mourners.

It is now when the mourners can see the defenses that no longer serve as avoidance, rationalization, rumination, and they can advance on the path of grief.

***“It distressed me a lot, and these days I feel that I have moved toward acceptance; we have all worked hard. I have learned from myself that I can express more.”***

### Unfinished Business

Unfinished business with whom we have lost makes separation difficult. Guilt, resentment, doubts act as invisible threads that bind parents and prevent farewell. As a result, energy is dispersed in the past, and parents look back and do not face the present in full potential.

The words that define this topic are guilt, accept, why, time, understand.

Guilt is one of the emotions that are most associated with grief and sadness. It often expresses itself in different ways. It can be felt toward oneself or someone else. In this case, it is closely connected to impotence since parents feel that they have not been able to defend their children. They also feel that they have failed to fulfill the primary function of the parent: protection.

***“I often feel guilty because I want her here with me, I want to touch her, and I can’t and impotence kills me and I feel much anger. Then I think about how bad she was, the suffering caused by the disease, and I feel very selfish to love her like that with me.”***

Accepting is the end that everyone wants to reach and feels that they cannot.

For each parent, the word “acceptance” has a different nuance but a common goal: to learn to live again.

***“Acceptance is to understand and believe that you can live with the absence you have left.”***

Death in children is seen as an event against nature, since it is not possible to fit in a logical order, and therefore, it is not understood.

An infinite range of “whys” and “what ifs” opens up until it becomes a labyrinth that traps parents, preventing progress in bereavement.

***“The more time passes, the more I feel bad, the bad thoughts do not go away and only the traumatic images come to me.”***

The concept of time is something complicated to explain for the mourners because the future cannot be thought of and the past is a continuous remembering and recalling trying to understand and retain. When parents feel that the months go by and instead of mitigating the pain increases, the fear of being “sick” grows, of never getting better, of being condemned to a life of suffering.

All the systems of certainties and logic that sustained each one’s life collapse during grief and “understanding” requires much effort. They work to understand all these answers so that they can close back on unfinished business and focus on new tasks.

***“I want to isolate myself because nobody understands it and people say non-sense, and for this reason, I prefer not to speak. In the group, I feel comfortable and understood.”***

## Discussion

The topic analysis provides interesting indications about the different interpretive journeys of the bereavement situation and offers ideas regarding the different types of social responses. The study and analysis of the speech of bereaved parents help us to understand the strategies that parents use to go through the grieving process, which goals and objectives are proposed in both the long and short term, and which tools they use in order to face the grief. This analysis helps both professionals and mourners to understand what the new construction of identity is. How the meaning resulting from the bereavement process and what are the categories that sustain it, some being linked to others, some independent, and some that end when others appear.

One of the most recognized benefits of group therapy is the creation of a space for the expression of emotions (Heiney et al., 1995). At this time, it is appreciated that experiencing pain involves allowing yourself to feel the full range of feelings and thoughts that accompany a loss, including sadness, anger, fear, anxiety, shame, guilt, and even relief. Although emotions are indeed forces that push us, shake us and wobble, it is also true that we can regulate them: feed them, understand them, express them, avoid them, and go through them. On the other hand, emotions also have an essential informative function. They tell us if our needs are being met or not, if we are respecting our values, if our ties are in danger.

As it has been possible to verify in this study, the topics that we mainly faced in the initial phase of the group have been remembering the most important events in the children’s lives; reconstructing the circumstances of death and illness; sharing emotions and coping mechanisms.

The emotional bereavement state is not a linear progression, it is instead a constant adaptation to an emotional roller coaster that has no logic or temporality. In addition to the value of emotional awareness as a source of information, symbolizing emotion in awareness promotes reflection on the experience to create new meaning, which helps patients develop new narratives to explain their experience (Greenberg, 2012).

Bereaved families often struggle against double isolation: one that is voluntary, since they are afraid of burdening their support network with their persistent sadness; and the other that is forced, since they perceive that social support diminishes over time (O’Connor and Barrera, 2014; Ljungman et al., 2015). Throughout group psychotherapy, parents once again trust people, open up again with others, share because they no longer feel “out of place.”

The therapeutic process begins with becoming aware of the defense mechanisms that are used against the suffering caused by loss. These are unconscious behaviors that allow us to defend ourselves from the threats that we perceive in our integrity as people, in our values and serve to avoid facing something that cannot be integrated or that feels intolerable. During therapy, tools are learned to know and manage the defensive mechanisms, to normalize them if they are adaptive and to change them if they are not helping mourners.

A relatively common source of discomfort and anxiety for grieving individuals is represented by unfinished business, as highlighted by the few empirical research on this construct (Steinhauser et al., 2015). In addition, different studies have shown the correlation between the presence of unfinished business with prolonged grief and other psychological and psychiatric disorders (Horowitz et al., 1993; Klingspon et al., 2015; Holland et al., 2020). Therefore, one of the most important tasks that can be done in bereavement is to help patients deal with their unfinished business, on which they are stuck or blocked.

Possible emotional responses to unfinished business can include various reactions, such as regret, anger, guilt, or remorse (Klingspon et al., 2015). Guilt deserves special mention since it is one of those responsible for the failure to close the bereavement and one of the most studied in this area Grinberg (1971). An important aspect of directing the work is to identify if there is regret behind the guilt. It represents a strong link with unfinished business (Klingspon et al., 2015) and it is emotionally related to unfavorable responses to one or different decisions made during treatments. Understanding the cause of death, talking about the course of illness and treatment, rebuilding the last hours of one’s child’s life, has been denoted as facilitating aspects of bereavement (Meyer et al., 2006; Meert et al., 2007; Eggly et al., 2011).

Studies identified the presence of unfinished business as an unfavorable cause for adaptive grief. Klingspon et al. (2015) emphasize that the presence of unfinished business shows worse results in bereavement, as indicated by more severe prolonged grief symptoms, significant psychiatric symptoms, and lack of construction of meaning regarding loss. In this study, we have seen how unfinished business are kept in a last and desperate attempt not to face the emptiness of loss, to remain stuck where we are, even if it is a place full of pain from the fear of letting go.

Another aspect that we have to pay attention to is what we call “legacy.” The use of legacy, of the continuous bonds with the deceased as a mechanism of adaptation to grief, is a new aspect and still little studied. The most influential authors (Klass et al., 1996; Currier et al., 2006; Neimeyer et al., 2006) do not reach the same conclusions about the usefulness of this construct for an adaptive bereavement process.

Klass (1997) confirm that the persistence of a strong bond with the deceased relative helps a reasonable resolution of the mourning; while authors such as Lehman et al. (1987) and Stroebe et al. (2012) question that a continuous bond may be an aspect that favors bereavement, and they believe that in some cases it may even make mourning work difficult. Currier et al. (2006) and Neimeyer et al. (2006) refer that the adaptive or maladaptive nature of the legacy depends on the way it is used by the bereaved. The disparity of opinions is explained in the light of the two meanings that can be attributed to the concept of a continuous bond. Connections with the loved one may be physical, such as having an object that serves as a memory. These rituals are defined as external links, prevalent in an immature and initial phase of bereavement and they are characterized by the inability to accept the physical absence of the loved one (Field et al., 2013; Zhang and Jia, 2018). While internalized forms can be sharing anecdotes, having lessons to follow, or feeling support in vital moments. In this sense, we refer to legacy, and we interpret it as an adaptive way of coping with grief (Field et al., 2005; Schaefer et al., 2020).

In this study, we showed how the individuation of an evident legacy with the child turns out to be adaptive and favorable for the parents. Many parents claim that their lives are guided by what was essential to their children. They describe living as the way to do things that their children valued or that their children had not been able to do. If parents manage to find the legacy that unites them with their children, they will be able to access a new sense of meaning.

It is important to note that parents struggle to make sense of their child’s loss, and those who are unable to do so are at greater risk for symptoms of prolonged grief; while the creation of meaning is associated with a better adjustment (Keesee et al., 2008; Lichtenthal et al., 2019; Grassi et al., 2021).

Gillies and Neimeyer (2006) propose a specific model for the reconstruction of meaning. The creating meaning process happens through three specific ways: the search for meaning, the search for benefits, and the identity change. Some families use religious and spiritual beliefs as a framework to make sense of their loss and find meaning, both in their lives and in those of the deceased (Folkman, 2001). In this study, we have seen how spirituality for some, covers an important role in achieving a new meaning to loss.

Various processes that make up the reconstruction of meaning seem to mitigate the impact of other risk factors focused on the characteristics of the individual grieving, the relationship with the deceased, and the death itself (Neimeyer et al., 2006).

Keesee et al. (2008) found that 47% of grieving parents were unable to create any meaningful sense of their loss for an average of 6 years. In other studies, many parents emphatically express that the loss of a child does not have a sense (Lichtenthal and Breitbart, 2015). In accordance with our study, these results would explain why the construction of meaning, even being helpful to the bereaved, is not easily achieved or accepted and explain why we had to work on the topic of “sense” the “reconstruction of meaning.”

In this area, it should be noted that this is a delicate issue to work with parents who, understandably, may have a difficult time identifying any positive outcomes that are associated with their loss.

As it was to be expected in this study, we had also encountered resistance when it was questioned whether we could find meaning in the death of children or could cause a positive movement in their lives. When explaining the topic to parents, they have recognized their work within this category and affirm that it is a complicated and laborious step, since it confronts them with the enormous turn their lives have taken; and in some cases, they are reluctant to get rid of the pain and of a new form of happiness.

### Limitations

We should pay attention to some limitations of this study. Firstly, it has intrinsic limitations to transcriptions there need to be very detailed and reflect tones of voice, inclinations, pauses that are sometimes difficult to write down. The results were triangulated to face this limitation, confirming them with the existing bibliography and carrying out different Focus Groups (Moriconi and Cantero-García, 2022) with the parents who participated in the therapeutic groups to discuss the findings. Second, it is important to note that the distortion factor can be given by the interpretation and subjectivity of the same researcher. Finally, it is essential to point out the limitations regarding the sample in terms of number and homogeneity.

### Conclusion

After reviewing the existing bibliography, we have confirmed the lack of specific literature on the problem of grief in parents whose children have died from cancer. Much research has shown that parents who lose a child to cancer want support, and there are still few studies on the most effective interventions for this group. Because of this scarcity, this work turns out to be a starting point and an incentive for future research.

Once we have analyzed the data related to the therapeutic sessions of the bereavement groups, we have been able to show five well-defined thematic nuclei that are well defined and not confluent with each other. We have named the topics Sense, Emotional Relief, Tools, Unfinished Business, and Legacy.

This study aims to help us understand the difficult mechanism that is set in motion in parents when a child dies, what they need and how it can be provided with effective and timely interventions. Health professionals who work with this group must bear in mind that each member of the family can experience and cope with loss in qualitatively different ways. Therefore, parents and close relatives need a well-defined and expertly guided space to be able to grieve. In addition, emphasis should be placed on the idea that there is not a “right” way to grieve, and the support must be built around individuals and what is useful during the specific grieving process.

The implications that this study has in clinical practice are useful not only in inherent bereavement in death, but also in the work of bereavement understood as a loss. Therefore, knowing the thematic nuclei on which the psychic elaboration of the trauma moves is relevant in all phases of the disease. Knowing the mechanisms underlying the development of loss prevents not only psychological suffering but also an increase in the human quality of care for patients.

## Data Availability Statement

The raw data supporting the conclusions of this article will be made available by the authors, without undue reservation.

## Ethics Statement

Ethical review and approval was not required for the study on human participants in accordance with the local legislation and institutional requirements. The patients/participants provided their written informed consent to participate in this study.

## Author Contributions

VM: conceptualization, methodology, investigation, software, data curation, and writing—original draft preparation. MC-G: visualization, supervision, and writing—reviewing and editing. Both authors have read and agreed to the published version of the manuscript.

## Conflict of Interest

The authors declare that the research was conducted in the absence of any commercial or financial relationships that could be construed as a potential conflict of interest. The reviewer JG-M declared a shared affiliation with one of the authors, MC-G, to the handling editor at the time of review.

## Publisher’s Note

All claims expressed in this article are solely those of the authors and do not necessarily represent those of their affiliated organizations, or those of the publisher, the editors and the reviewers. Any product that may be evaluated in this article, or claim that may be made by its manufacturer, is not guaranteed or endorsed by the publisher.
